# Atomistic mechanisms underlying the activation of the G protein-coupled sweet receptor heterodimer by sugar alcohol recognition

**DOI:** 10.1038/s41598-019-46668-w

**Published:** 2019-07-15

**Authors:** Panupong Mahalapbutr, Nitchakan Darai, Wanwisa Panman, Aunchan Opasmahakul, Nawee Kungwan, Supot Hannongbua, Thanyada Rungrotmongkol

**Affiliations:** 1 Structural and Computational Biology Research Unit, Department of Biochemistry, Faculty of Science, Chulalongkorn University, Bangkok, 10330 Thailand; 2 Program in Biotechnology, Faculty of Science, Chulalongkorn University, Bangkok, 10330 Thailand; 3 Multidisciplinary Program of Petrochemistry and Polymer Science, Faculty of Science, Chulalongkorn University, Bangkok, 10330 Thailand; 4 Computational Chemistry Center of Excellent, Department of Chemistry, Faculty of Science, Chulalongkorn University, Bangkok, 10330 Thailand; 5 Department of Chemistry, Faculty of Science, Chiang Mai University, Chiang Mai, 50200 Thailand; 6 Center of Excellence in Materials Science and Technology, Chiang Mai University, Chiang Mai, 50200 Thailand; 7 Ph.D. Program in Bioinformatics and Computational Biology, Faculty of Science, Chulalongkorn University, Bangkok, 10330 Thailand; 8 Molecular Sensory Science Center, Faculty of Science, Chulalongkorn University, Bangkok, 10330 Thailand

**Keywords:** Computational models, Theoretical chemistry

## Abstract

The human T1R2-T1R3 sweet taste receptor (STR) plays an important role in recognizing various low-molecular-weight sweet-tasting sugars and proteins, resulting in the release of intracellular heterotrimeric G protein that in turn leads to the sweet taste perception. Xylitol and sorbitol, which are naturally occurring sugar alcohols (polyols) found in many fruits and vegetables, exhibit the potential caries-reducing effect and are widely used for diabetic patients as low-calorie sweeteners. In the present study, computational tools were applied to investigate the structural details of binary complexes formed between these two polyols and the T1R2-T1R3 heterodimeric STR. Principal component analysis revealed that the Venus flytrap domain (VFD) of T1R2 monomer was adapted by the induced-fit mechanism to accommodate the focused polyols, in which residues 233–268 moved significantly closer to stabilize ligands. This finding likely suggested that these structural transformations might be the important mechanisms underlying polyols-STR recognitions. The calculated free energies also supported the VFD of T1R2 monomer as the preferential binding site for such polyols, rather than T1R3 region, in accord with the lower number of accessible water molecules in the T1R2 pocket. The E302 amino acid residue in T1R2 was found to be the important recognition residue for polyols binding through a strongly formed hydrogen bond. Additionally, the binding affinity of xylitol toward the T1R2 monomer was significantly higher than that of sorbitol, making it a sweeter tasting molecule.

## Introduction

**Figure 1:**
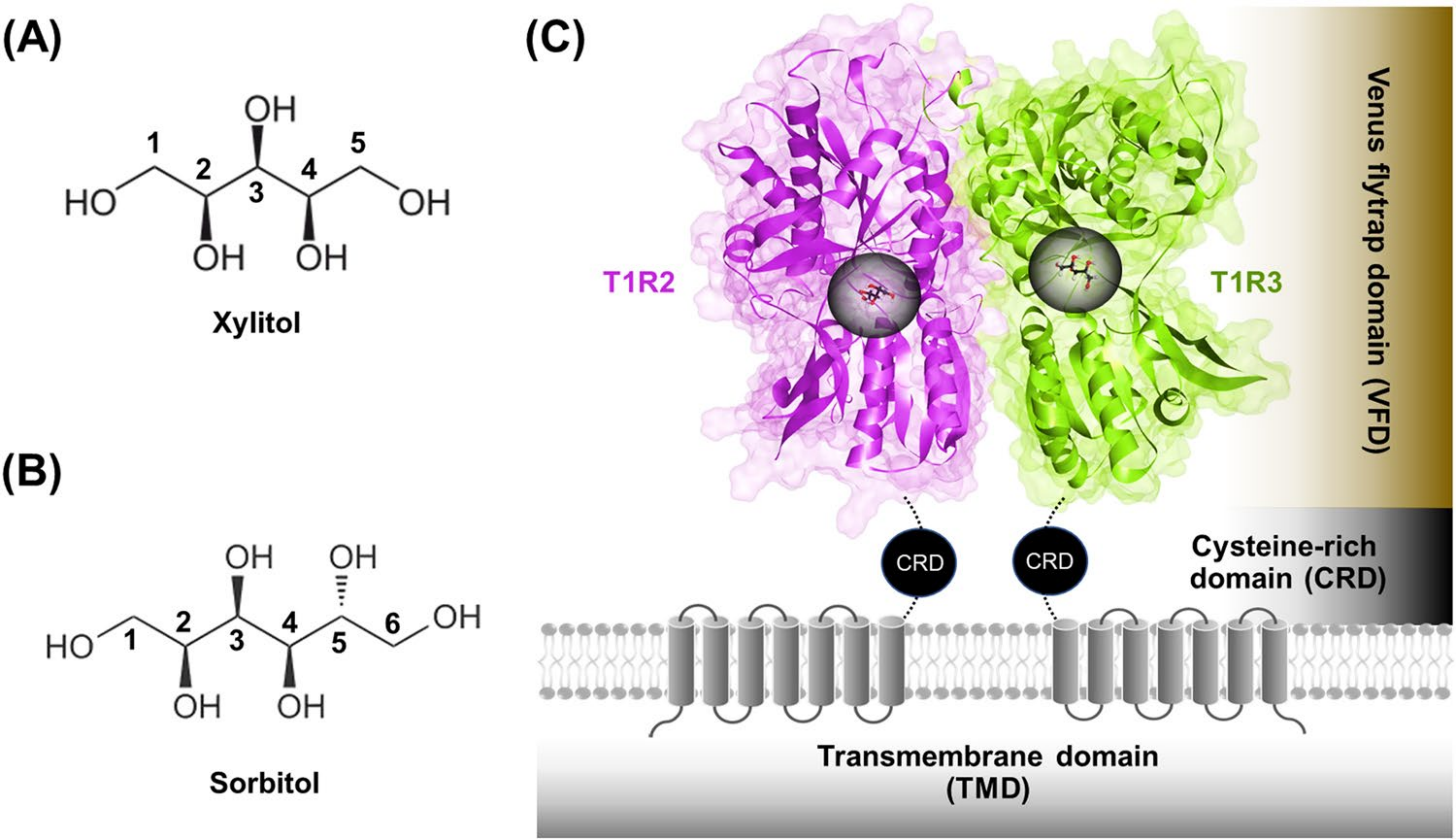
Chemical structure of (**A**) xylitol and (**B**) sorbitol. (**C**) The T1R2 (pink) and T1R3 (green) monomers forming a heterodimeric STR, in which the portions of the VFD, CRD, and TMD are shaded by brown, black, and grey, respectively. The ligand-binding pockets for both the T1R2 and T1R3 monomers are represented by black circle.

The increased intake of processed food containing a high level of sugars is likely to be a leading cause of deleterious health effects, ranging from inflammation to obesity, type 2 diabetes, and coronary heart disease^1–4^. Accordingly, the use of alternative sweeteners with a low calorific value has increased recently in an attempt to reduce sugar (and calorie) consumption so as to avoid such diseases. About 100 y ago, saccharin and cyclamates were introduced to the marketplace as artificial sweetening agents with about 300- and 40-fold greater, respectively, sweetening capacity than sucrose^5,6^. However, such sweeteners have since been indicated as cancer-promoting agents^7,8^. Xylitol and sorbitol (Fig. 1A,B) are representative sugar alcohols (also known as polyols) that are naturally found in many fruits and vegetables and can serve as an alternative less controversial sweetener with a caries-reducing effect^9^. They are widely utilized in the diet of diabetic patients as low-calorie sweeteners^10^. In terms of cariogenesis, xylitol and sorbitol do not decrease the plaque pH to a point where enamel demineralization occurs, and so this leads to reduced plaque accumulation^11^. Interestingly, there is no evidence that xylitol increases the triglyceride and blood glucose levels^10^.

Sweet sensing in the tongue is mediated by the human sweet taste receptor (STR), which belongs to the class C G protein-coupled receptor (GPCR) family, and is composed of the taste 1 receptor (T1R) and the taste 2 receptor (T2R) families^12,13^. Two subtypes of the T1R family, T1R member 2 (T1R2) and T1R member 3 (T1R3), form a heterodimer (T1R2-T1R3; Fig. 1C) to act as a STR. The T1R2-T1R3 heterodimeric STR possesses a large extracellular Venus flytrap domain (VFD), which is the binding site of sweet-tasting ligands, linked to an α-helical transmembrane domain (TMD) by a short cysteine-rich domain (CRD). The T1R2-T1R3 STR expressed on the surface of the tongue^14^ can be activated by a broad range of sweet-tasting molecules, including sugars (monosaccharides and disaccharides), artificial sweeteners (saccharin and cyclamates), amino acids (tryptophan, serine, and phenylalanine)^12^, small sweet-tasting proteins (thaumatin, monellin, brazzein, and neoculin)^15^, and sugar alcohols (sorbitol and xylitol)^16,17^. The activated T1R2-T1R3 receptor triggers the downstream signaling cascades, including the dissociation of the heterotrimeric G protein (α-gustducin, Gβ3, and Gγ13), leading to the release of intracellular Ca^2+^ and the ATP exocytosis, which in turn activates purinergic receptors on afferent fibers and results in taste perception^12,18^.

The X-ray crystal structure of the VFD of the homodimeric metabotropic glutamate receptor subtype 1 (mGluR1) has been identified, representing the first structure of a class C GPCR^19^. The amino acid sequences of the VFD of T1R and mGluR1 exhibit ~25% identity and ~41% similarity^20^. Furthermore, the predicted secondary structures (helices and β-pleated sheets) of the VFD of T1R match to those of mGluR1, suggesting that T1R2-T1R3 heterodimer could recognize the ligand in a manner similar to mGluR1^20,21^. Although the structural details as well as the mechanisms underlying the activation of STR by small molecule sweeteners and proteins have been investigated^15,22,23^, the structural dynamics, intermolecular interactions at the atomic level, and the preferential binding site of the two polyols, xylitol and sorbitol, toward STR remain largely unexplored.

This research aimed to theoretically investigate the mechanisms by which the human T1R2-T1R3 heterodimeric STR is activated by the naturally occurring polyol sweeteners, xylitol and sorbitol, so as to understand the dynamics behavior and atomistic details of such binary complexes. Moreover, the preferential binding site and the key binding residues for these polyols were also characterized.

## Results

**Figure 2:**
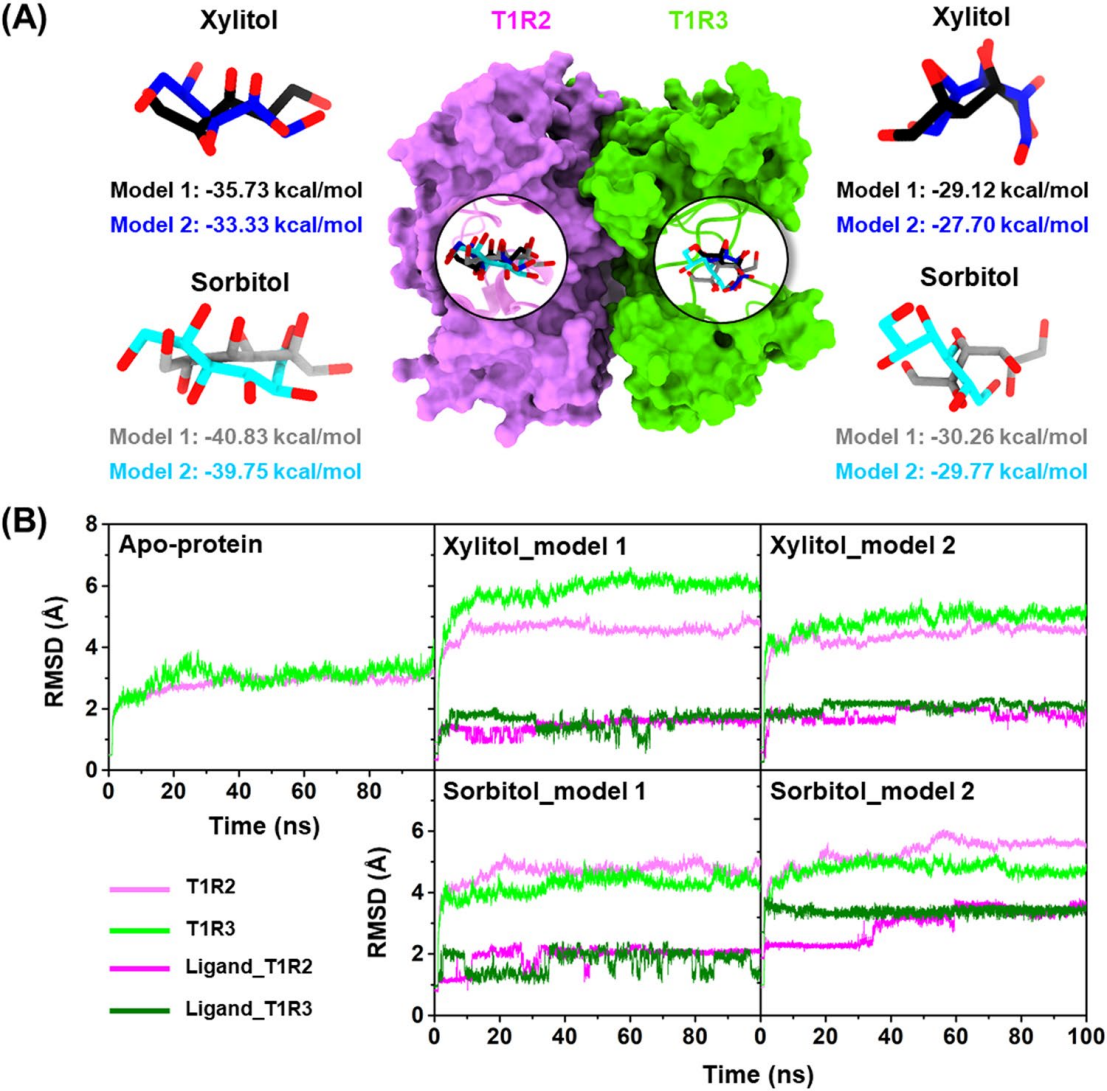
(**A**) Superimposed docked structures between model 1 and model 2 of xylitol and sorbitol in complex with T1R2 (pink) and T1R3 (green) monomers, where the CDOCKER interaction energies (kcal/mol) are given below. (**B**) All-atom RMSD plots for the T1R2-T1R3 heterodimeric STR in (**left**) apo form or with (**middle** and **right**) xylitol or sorbitol bound at the two glutamate-binding sites.

Triplicate molecular dynamics (MD) simulations of two different initial docked structures (Fig. 2A), namely model 1 and model 2, which showed respectively the first- and second-lowest CDOCKER interaction energies, provided somewhat similar results. Therefore, only qualitative data taken from one replication of each model are represented here for simplification, whereas the quantitative results were averaged from three independent replications (shown as mean ± standard error of mean (SEM)).

### System stability

The stability of MD systems was analyzed using root-mean-square displacement (RMSD) calculations. As shown in Fig. 2B and Supplemental Fig. S1, the RMSD values for T1R2 (pink) and T1R3 (green) monomers of all systems relative to those of starting structures rapidly increased during the first 50 ns and then remained at a fluctuation of ~3.0–4.0 Å and ~4.0–6.0 Å for the apo-protein and ligand-bound forms, respectively. Enhanced RMSD values in both monomers of heterodimeric STR due to ligand binding were detected, indicating that ligand recognition caused the T1R2-T1R3 conformational changes (discussed in more detail later). For these two polyols, the RMSDs of xylitol and sorbitol showed similar patterns at both the T1R2 (magenta) and T1R3 (dark green) monomers, where the RMSD increased in the first 5 ns and then remained at ~1.5–2.0 Å until the end of simulation. In addition, xylitol showed a higher level of fluctuation at T1R3 monomer than T1R2, implying that xylitol binds well within the T1R2 region.

In this work, the last 30-ns trajectories of the 100-ns MD simulations of each system were extracted for further analysis in terms of the: (*i*) essential dynamics of the protein upon polyols complexation by principal component analysis (PCA), (*ii*) binding affinity of the sugar alcohols based on the molecular mechanics (MM) combined with the Poisson-Boltzmann (PB) or generalized Born (GB) surface area (MM/PB(GB)SA) calculations, (*iii*) key binding residues by per-residue decomposition free energy (Δ*G*_bind,res_) calculations, (*iv*) ligand-protein hydrogen bonding, and (*v*) water accessibility toward the binding pocket.

### Essential dynamics of the T1R2-T1R3 STR upon polyols complexation

**Figure 3:**
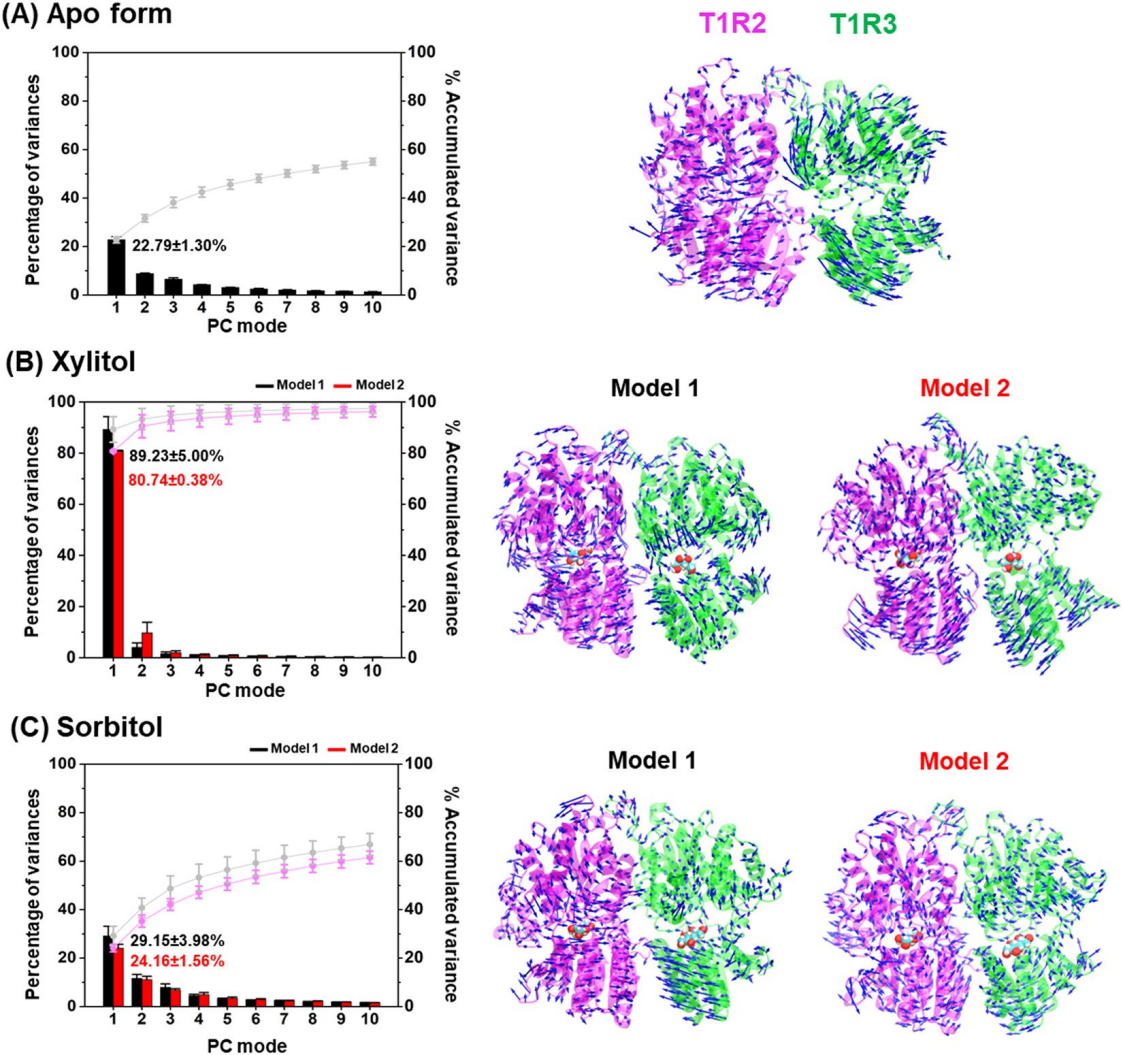
(**Left**) The quantitative scree diagram and (**Right**) its porcupine plot of the PC1 for the (**A**) apo-protein, (**B**) xylitol-STR, and (**C**) sorbitol-STR. Both xylitol and sorbitol are shown in van der Waals model.

**Figure 4:**
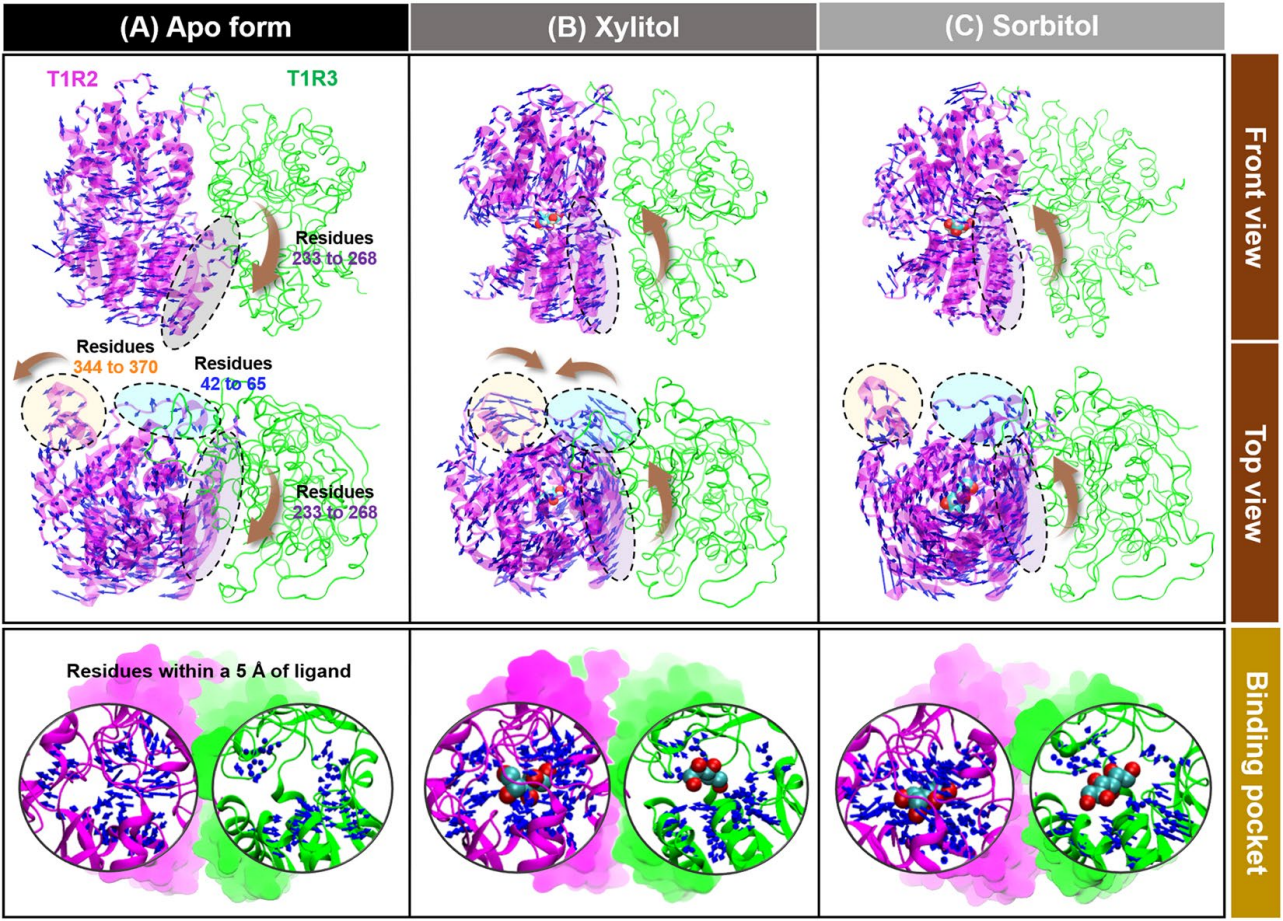
PC1’s porcupine plot of model 1 of the (**A**) apo-protein, (**B**) xylitol-STR, and (**C**) sorbitol-STR showing the significant motions of (*i*) residues 42–65 (blue dashed circle), (*ii*) residues 233–268 (purple dashed circle), (*iii*) residues 344–370 (orange dashed circle), and (*iv*) residues within 5 Å of the respective ligand (black circle) across the PC1.

The structurally relevant motions of both the apo and holo forms of T1R2-T1R3 heterodimeric STR were investigated using PCA^24^ on 1,000 snapshots extracted from the last 30 ns of MD simulations. The results of the first principal component (PC1) are illustrated in Fig. 3, while the focused regions are depicted in Fig. 4, in which the arrow and its length (blue) indicate the direction and amplitude of motions, respectively. Note that the PCA results of the apo form were independent, whilst those of xylitol- and sorbitol-bound forms were calculated along with the apo-protein using the same eigenvector, which could provide the crucial motions of the protein derived from ligand recognition.

The first 10 PC modes (Fig. 3, left) accounted for 54.97 ± 1.44%, 97.44 ± 1.87%, 96.18 ± 2.02%, 66.88 ± 4.53%, and 61.52 ± 2.62% of the accumulated variance for the apo-protein, xylitol-model 1, xylitol-model 2, sorbitol-model 1, and sorbitol-model 2, respectively. The percentage of variance of PC1 in each system was remarkably higher than that of PC2, suggesting that this mode can represent the crucial motions of the T1R2-T1R3 heterodimeric receptor.

Since quantitative PCA results from triplicate MD runs of model 1 and model 2 are somewhat similar, the PC1’s porcupine plot taken from one replication of each model was herein selected as a representative structure (Fig. 3, right). The results revealed that each sugar alcohol binding dramatically converted the direction of motions to point toward the ligands in both T1R2 and T1R3 monomers, in a manner different from that of the apo-protein. However, the overall protein conformation in the T1R3 monomer (green) after ligand binding was less compact than that in T1R2 portion (pink). In addition, the ligand-binding cavity of T1R3 for xylitol- and sorbitol-bound forms is as wide as that in apo-protein (Fig. 4, bottom panel), suggesting that T1R3 is not the preferential binding site for these two polyols. Accordingly, only T1R2 portion was selected for representing the atomistic mechanisms underlying the activation of STR.

In the apo form of the STR, the amino acid residues located within 5 Å of the ligand inside the T1R2 monomer were pointing outward from the ligand-binding site and were far away from each other (Fig. 4), representing the open binding pocket. Interestingly, xylitol and sorbitol bindings induced the adaptation of this pocket to regulate the ligand-protein complexation, in which these amino acid residues moved closer toward the ligands, resulting in an active conformation^25^. Moreover, binding of these polyols stimulated the direction of motion of residues 233–268 (purple dashed circle), located near to the binding pocket, to become significantly closer to xylitol and sorbitol, resulting in a compact molecular shape. Additionally, xylitol binding promoted residues 42–65 and 344–370 to point toward each other, making a closer packed structure than that in the sorbitol-STR complex (Fig. 4B, top panel).

### Binding affinity of the two polyols against the T1R2-T1R3 STR

**Figure 5:**
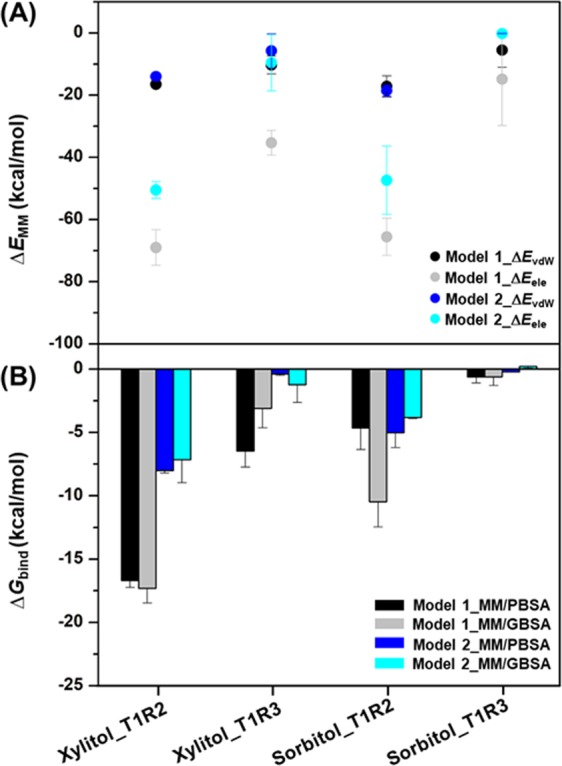
(**A**) The MM energy (kcal/mol) comprising the Δ*E*_ele_ and Δ*E*_vdW_ energy components. (**B**) The averaged MM/GBSA Δ*G*_bind_ (kcal/mol) of xylitol and sorbitol bindings to the T1R2-T1R3 heterodimeric STR. Data are expressed as mean ± SEM (n = 3).

To estimate the binding affinity of sorbitol and xylitol at the two binding sites of the T1R2-T1R3 receptor, the MM/PB(GB)SA methods were employed on the 100 snapshots taken from the last 30 ns of three independent MD simulations. The binding free energy (Δ*G*_bind_) results are shown in Fig. 5 together with its electrostatic (Δ*E*_ele_) and van der Waals (vdW; Δ*E*_vdW_) energy components. Note that the MM/PBSA and MM/GBSA results of model 1 and model 2 gave the similar trend; thus, only the free energies obtained from the MM/PBSA method of model 1 are shown (in parenthesis) below to simplify the interpretation.

In the gas phase, the MM energy showed that electrostatic interactions were the main stabilizing force for the sorbitol and xylitol bindings toward both the T1R2 (−69.01 kcal/mol for xylitol and −65.55 kcal/mol for sorbitol) and T1R3 (−35.35 kcal/mol for xylitol and −14.87 kcal/mol for sorbitol) monomers of the STR, and these were ~three- to five-fold higher than the vdW interactions. Most importantly, the Δ*G*_bind_ results strongly supported the VFD of the T1R2 monomer as the preferential binding site for both xylitol (−16.69 kcal/mol) and sorbitol (−4.66 kcal/mol) rather than the T1R3 region (−6.48 and −0.62 kcal/mol for xylitol and sorbitol, respectively). Furthermore, xylitol binding showed a significantly higher binding affinity (~four-fold for MM/PBSA and ~two-fold for MM/GBSA) than sorbitol at the VFD of the T1R2 monomer.

### Key binding residues

**Figure 6:**
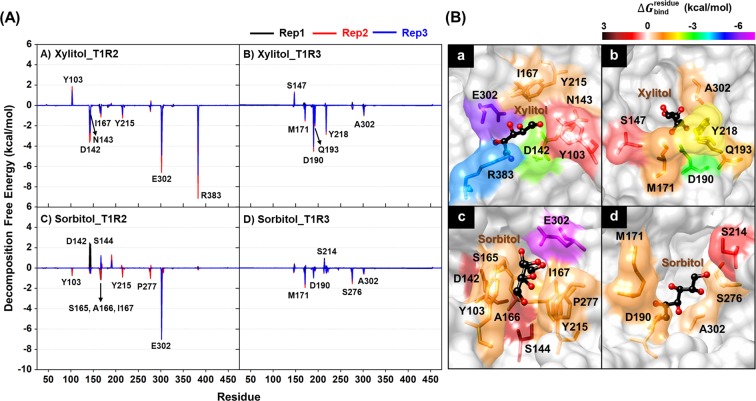
(**A**) Δ*G*_bind,res_ (kcal/mol) of T1R2 (left) and T1R3 (right) monomers for xylitol and sorbitol bindings based on MM/PBSA method. Black, red, and blue lines represent replications #1, #2, and #3, respectively. (**B**) The binding orientations of **(**a**)** xylitol-T1R2, **(**b**)** xylitol-T1R3, **(**c**)** sorbitol-T1R2, and **(**d**)** sorbitol-T1R3 complexes drawn from the last MD snapshot of replication #1 of model 1. The contributing amino acids involved in the ligand binding are colored according to their Δ*G*_bind,res_ values, where the highest to lowest free energies ranged from black to magenta, respectively.

The Δ*G*_bind,res_ calculation was employed to identify the key amino acids involved in ligand binding. The total contribution of each amino acid in T1R2 and T1R3 monomers for the two polyols are summarized in Fig. 6A, where the positive and negative Δ*G*_bind,res_ values represent the ligand destabilization and stabilization, respectively. The ligand binding orientations of each system (model 1) are illustrated in Fig. 6B, in which the contributing amino acids are colored according to their Δ*G*_bind,res_ values. Note that the Δ*G*_bind,res_ results obtained from replications #1-3 of model 1 (Fig. 6) and model 2 (Fig. S2) were almost identical; thus, only the data obtained from replication #1 of model 1 were discussed below for simplification.

In the case of xylitol, there were six (D142, N143, I167, Y215, E302, and R383) and five (M171, D190, Q193, Y218, and A302) amino acids involved in ligand binding at T1R2 and T1R3 monomers, respectively. The E302 (Δ*G*_bind,res_ of −5.74 kcal/mol, purple) and R383 (Δ*G*_bind,res_ of −4.47 kcal/mol, blue) residues located in the T1R2 monomer showed a strong binding affinity toward xylitol. For sorbitol, there were seven (Y103, S165, A166, I167, Y215, P277 and E302) and four (M171, D190, S276, and A302) residues contributed to ligand stabilization for the T1R2 and T1R3 monomers, respectively. Similar to xylitol binding, the sorbitol was predominantly stabilized by the E302 residue with a Δ*G*_bind,res_ of −6.57 kcal/mol (magenta) in T1R2 monomer. Taken together, the T1R2 monomer of human STR showed more contributing amino acids against polyols binding than T1R3, suggesting that this monomer is the preferential binding site for sorbitol and xylitol, in good agreement with the more intense PC1 arrows directed toward the ligand molecule in this region (Fig. 4).

### Ligand-protein hydrogen bonding

**Figure 7:**
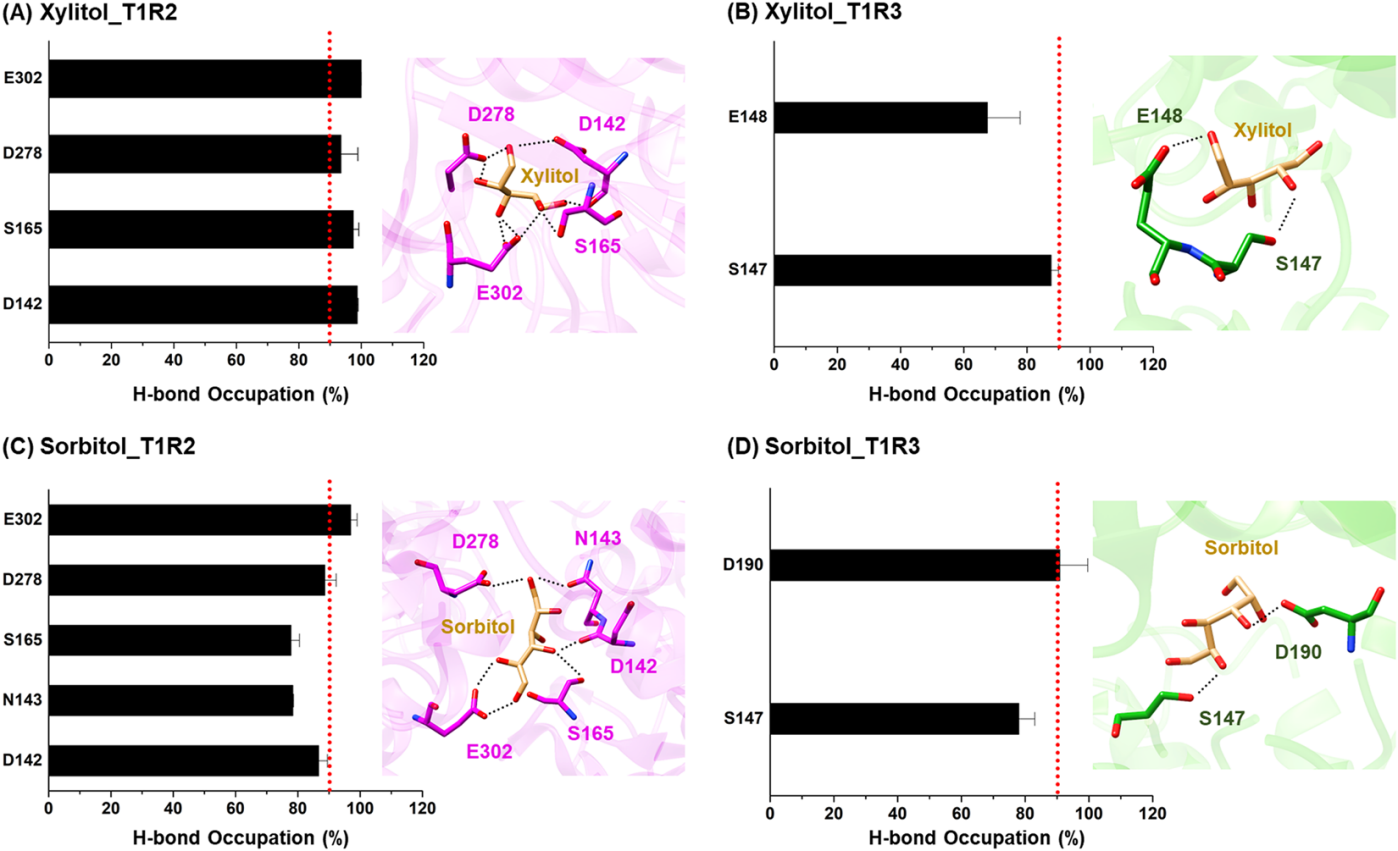
The averaged %HB_oc_ of the T1R2-T1R3 residues contributing to (**A**,**B**) xylitol and (**C**,**D**) sorbitol bindings over the last 30 ns of triplicate MD simulations. Red dash line indicates strong H-bond formation (**≥**90% occupancy).

Since electrostatic interactions were the main force driving protein-ligand recognition (Fig. 5), structural insights into hydrogen bond (H-bond) formation was then calculated using the defining criteria of: (*i*) the distance between H-bond donor (HD) and H-bond acceptor (HA) was ≤3.5 Å, and (*ii*) the angle of HD−H···HA was ≥120 degree. The averaged percentage of H-bond occupations (%HB_oc_) calculated from three independent MD simulations of model 1 are illustrated in Fig. 7, whereas the results of model 2 are depicted in Fig. S3. Note that only amino acids contributing to ligand binding with the %HB_oc_ of >50 were shown. As expected, high H-bonds (%HB_oc_ ≥70) were formed between the polar ligands and the surrounding charged residues inside T1R2-T1R3 heterodimeric STR. The number of H-bond formations found in T1R2 monomer (4–5) was higher than in T1R3 region (2), in good agreement with the Δ*G*_bind,res_ calculations described above (Fig. 6). The four (D142, S165, D278, and E302) and five (D142, N143, S165, D278, and E302) polar residues at T1R2 exhibited high %HB_oc_ with xylitol and sorbitol, respectively, whereas only three amino acids (S147, E148, and D190) at T1R3 formed H-bonds with polyols binding. Remarkably, the number of strong H-bonds (≥90% occupancy, red dash line) was more pronounced in xylitol-T1R2 system (4) than in sorbitol-T1R2 model (1), and the residue E302 of the T1R2 monomer showed the highest %HB_oc_ in both systems (99.9% for xylitol and 96.95% for sorbitol). In summary, the obtained H-bond information strongly supported the role of E302 residue in xylitol and sorbitol bindings toward the T1R2-T1R3 STR.

### Water accessibility in the binding pocket

**Figure 8:**
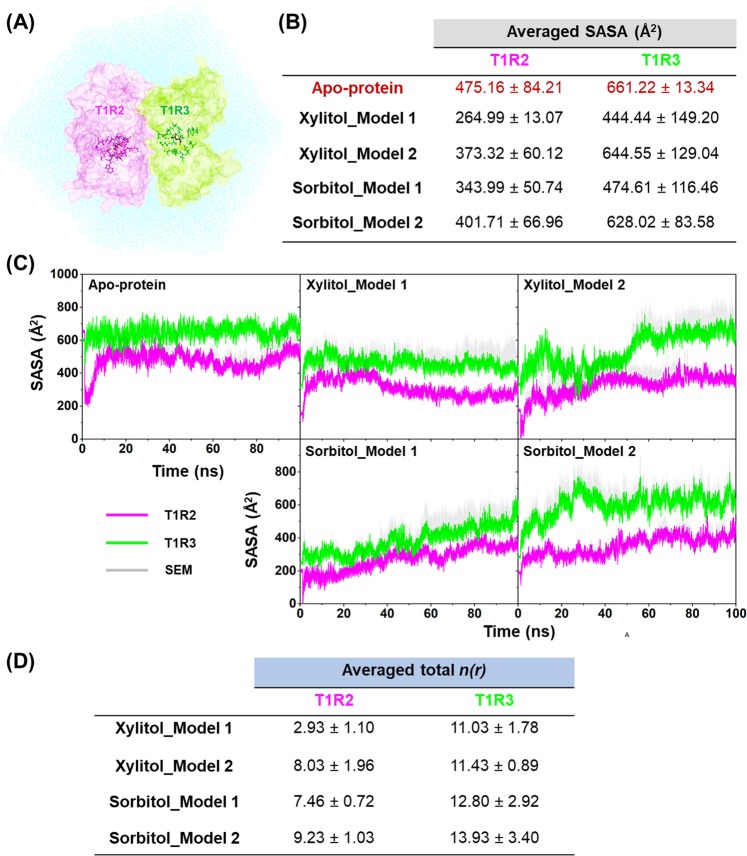
(**A**) The T1R2 (pink) and T1R3 (green) monomers comprising amino acid residues within 5 Å of ligands that were used for SASA calculations. (**B**) The averaged SASA (Å^2^) of the ligand-binding pockets in the T1R2 and T1R3 monomers calculated from the last 30 ns of three independent MD simulations. (**C**) The SASA (Å^2^) plot along the simulations. (**D**) The averaged total *n(r)* of all studied complexes. Data are expressed as mean ± SEM (n = 3).

The water accessibility in the ligand-binding pockets of T1R2 and T1R3 monomers was investigated using the solvent accessible surface area (SASA) calculations on the residues within a 5-Å sphere of each ligand (Fig. 8A–C). In addition, the radial distribution function (RDF; Figs S4 and S5) toward all oxygen atoms of xylitol (O1, O2, O3, O4, and O5; Fig. 1A) and sorbitol (O1, O2, O3, O4, O5, and O6; Fig. 1B) was used to characterize the number of water molecules approaching the ligands (Fig. 8D). Since model 1 and model 2 exhibited similar trend, only SASA results of model 1 were discussed below.

In the case of apo-protein, the averaged SASA values calculated from the last 30-ns simulations were 475.16 Å^2^ and 661.22 Å^2^ for the T1R2 and T1R3 monomers, respectively. Upon molecular complexation with the two polyols, the SASA values of both the T1R2 and T1R3 monomers were dramatically decreased, in which the SASA values of xylitol model (264.99 Å^2^ for T1R2 and 444.44 Å^2^ for T1R3) were lower than those of the sorbitol system (343.99 Å^2^ for T1R2 and 474.61 Å^2^ for T1R3). According to the RDF results, the averaged values of total integration number (*n(r)*), which describes the number of water molecules approaching the given oxygen atoms of each respective ligand, were considerably higher in T1R3 monomer (11.0–12.8) than in the T1R2 region (2.9–7.5). Furthermore, the *n(r)* of xylitol binding (2.9) was significantly lower than that of sorbitol (7.5) at T1R2 region (Fig. 8D).

## Discussion

In this work, the molecular modeling approaches were used to derive the structural insights into binary complexes formed between two polyols (xylitol and sorbitol) and the human T1R2-T1R3 heterodimeric STR. As *(i)* xylitol and sorbitol preferentially bind to VFD of STR rather than to TMD and CRD (Table S1) and *(ii)* the structural adaptation of VFD induced by sugar alcohols has not yet been fully revealed, we, therefore, focused on only VFD for investigating the atomistic binding mechanisms upon polyols recognition.

By considering the PCA, the porcupine plot of PC1 mode suggested that the VFD of T1R2 was the favorable binding site for both polyols, since the overall protein conformation in the T1R2 monomer was denser than that in the T1R3 region. Moreover, the ligand-binding cavity of T1R3 was as wide as that in the apo form. These structural transformations are in accord with the fact that the VFD of T1R2 is a recognized site for low-molecular-weight sugars, whereas the T1R3 monomer is proposed for the binding of sweet-tasting proteins, such as brazzein and neoculin^23,26–28^. Furthermore, binding of the two polyols converted the direction of motion of not only amino acids within a spherical radius of 5 Å but also residues 233–268, which are near to the binding pocket, to become significantly closer to the ligands. This was especially the case for xylitol, which caused residues 42–65 and 344–370 to point toward each other, resulting in a more compact shape than that with sorbitol binding. This could be the reason why the binding affinity of xylitol toward T1R2 was significantly greater than that of the sorbitol-T1R2 complex (Fig. 5).

Previous studies revealed that the bindings of mogroside V, a nonstevia glycoside, and aspartame resulted in the closed form of the T1R2 monomer as compared to the open form of apo-protein^25,29^. However, the atomistic insights and dynamics behavior of polyols-STR complexes have not yet been fully revealed. Accordingly, this is the first time that we could shed light on the atomistic adaptation of T1R2 mediated by xylitol and sorbitol, in which they utilize the induced-fit mechanism to regulate the activation of G protein-coupled STR. This finding was supported by MM/PB(GB)SA calculations demonstrating that the T1R2 monomer was the suitable site for the binding of these two polyols rather than T1R3 monomer. Moreover, xylitol binding showed a significantly higher binding strength than sorbitol toward the T1R2 monomer. Thus, the obtained free energy differences as well as the structural adaptations (taken from PCA) might be the reason why xylitol tastes much sweeter than sorbitol^10^. From the evaluation of MM energy, electrostatic energy was the main force inducing receptor activation, being about three- to five-fold higher than vdW interactions, which agreed well with previous studies on the small-molecule sweeteners, thaumatin and cyclamate, and sweet proteins^30–32^.

The Δ*G*_bind,res_ calculations revealed that there were six (D142, N143, I167, Y215, E302, and R383) and seven (Y103, S165, A166, I167, Y215, P277 and E302) amino acid residues in the T1R2 monomer associated with xylitol and sorbitol bindings, respectively. The importance of residues Y103, D142, S165, Y215, E302, and R383 in the T1R2 monomer correlated well with the reported binding of several sugar derivatives at VFD of T1R2 monomer^21,23,33^, suggesting that these residues play a crucial role as recognition sites for low-molecular-weight sweeteners. Apart from the electrostatic attractions, H-bond formation is one of the important factors determining the protein-ligand stability. Our calculation showed that residue E302 of the T1R2 monomer exhibited the strongest H-bond formation (almost 100% occupancy) in both xylitol and sorbitol bindings along the last 30 ns of simulations. This result strongly agreed with previous studies on the other ligands binding to the STR, where the negatively charged E302 residue promotes H-bond formation with sucralose and saccharin at the VFD of T1R2^23,27^. Furthermore, E302 has been reported as a crucial residue for aspartame binding^26^, since the E302A mutation dramatically diminishes the half-maximal effective concentration (EC_50_) value of aspartame^23^.

In the case of ligand binding mode, it was reported that the T1R2 residues D142, R383, Y103, and S165 interacted respectively with the backbone oxygen atoms, carboxyl group, methyl moiety, and phenyl ring of aspartame^34^. Additionally, the oxygen atoms of sucralose were mainly stabilized by Y103, D142, D278, and E302^23^. In correlation with these reports, our present study showed that the residues Y103, D142, S165, and E302 of T1R2 monomer were involved in xylitol and sorbitol bindings (Fig. 6A,B), indicating that these two polyols share a similar binding pattern to the other sweet tasting molecules. Taken together, our MM/PB(GB)SA-based free energies and H-bond calculations could successfully predict the important amino acids associated with ligand binding.

The conformational changes of T1R2-T1R3 STR derived from polyols binding might alter the water accessibility toward ligand-binding pocket in a manner different from that of apo form, which could importantly affect the protein-ligand interactions. Thus, the SASA and RDF calculations were employed to evaluate this possibility. The acquired results suggested that the T1R2 monomer was the preferential binding site for these two polyols rather than T1R3 region, since a low water accessibility was observed in the T1R2. In contrast, a large number of water molecules in the binding pocket could considerably affect the protein-ligand binding affinities in T1R3 monomer by interfering with the electrostatic interactions (Fig. 5), resulting in a lower binding efficiency. Interestingly, the number of water molecules detected in xylitol-T1R2 complex was significantly lower than that in the sorbitol-T1R2 complex, suggesting that the molecular structure of xylitol fits better within the ligand-binding pocket of T1R2 than sorbitol. In summary, the solvation calculations totally agreed with the MM/PB(GB)SA and PCA results.

In conclusion, this work provided useful structural details of the two polyols (sorbitol and xylitol) bound to the T1R2-T1R3 STR. Most importantly, this is the first report to show that xylitol and sorbitol atomistically adapt the conformation of STR to become a close-packed structure through an induced-fit mechanism. In addition, the preferential binding site and the key binding residues involved in sorbitol and xylitol bindings were revealed, which can be further used as a rational guideline for designing and developing new agonist sugar alcohol derivatives against the T1R2 monomer of human STR.

## Materials and Methods

### Preparation of initial structures

The nucleotide sequences of human STR T1R2 (GenBank: BK000151) and T1R3 (GenBank: BK000152) were obtained from the National Center for Biotechnology Information (NCBI). The homology model of the heterodimeric T1R2-T1R3 STR was constructed by the SWISS-MODEL server^35^ using the crystal structure of mGluR1 (PDB ID: 1EWK)^19^ as a template. The protonation state of all ionizable amino acid residues was characterized using PROPKA 3.0^36^ at pH 7.0. The starting structures of xylitol and sorbitol were built and fully optimized by the HF/6-31 G* level of theory using Gaussian09 program^37^.

### Preparation of polyols-STR complexes

Xylitol and sorbitol were separately docked with 100 docking runs into the two glutamate-binding sites (spherical radius of 10 Å, Fig. 1C) of T1R2-T1R3 heterodimer using CDOCKER module^38^ implemented in Accelrys Discovery Studio 2.5^Accelrys Inc.^. The docked complexes with the first- (model 1) and second-lowest (model 2) interaction energies for each ligand binding at T1R2 and T1R3 monomers were chosen as the starting structure for further investigations in comparison to the apo form of T1R2-T1R3. In order to evaluate the reliability of the generated ligand-protein complex derived from CDOCKER, FlexX docking program was applied to calculate the binding selectivity of polyols toward three domains of STR (Table S1). The electrostatic potential (ESP) charges were calculated on the optimized structure of ligand by the HF/6-31 G* level of theory as per the standard procedures^39–42^. The antechamber implemented in AMBER14 was used to generate the restrained ESP (RESP) charges of the ligand. The AMBER ff12SB^43^ and the general AMBER force field^44^ were applied for protein and ligands, respectively. Note that the RESP charges and MM parameters used for the ligands were given in Supplementary Information (Tables S2–5). Missing hydrogen atoms were added using the LEaP module. Subsequently, each system was solvated in the TIP3P water^45^ with a distance of 12 Å from the protein surface, and the Na^+^ ions were then randomly added for neutralizing the system. The added hydrogen atoms and water molecules were minimized using 3,000 steps of steepest descents and switched to 2,500 steps of conjugated gradient minimization process. Finally, the whole system was fully minimized using the same methods.

### MD simulations, structural analyses, and free energy calculations

MD simulations on the 15 systems (T1R2-T1R3 STR without (3) and with either xylitol (6) or sorbitol (6) bound) were performed under a periodic boundary condition using AMBER 14 program. The entire covalent hydrogen bonds were constrained using the SHAKE algorithm^46^. A short-range cutoff of 10 Å was employed for non-bonded interactions, while the Particle Mesh Ewald (PME) summation method^47^ was applied to treat the long-range electrostatic interactions. The systems were heated up to 310.0 K for 100 ps. Afterward, the simulations with *NPT* ensemble were performed at this temperature until the simulation time reached 100 ns. The cpptraj module was used to compute the structural analyses as follows.

The equilibrium state of all simulated models was determined by computing the RMSD. The PCA and H-bond calculations were used to investigate the relevant motions and structural details of the studied complexes, respectively. The SASA and RDF were employed to characterize the water accessibility at ligand-binding pocket of both T1R2 and T1R3 monomers. Moreover, the MM/PB(GB)SA binding free energy calculations^48^ were performed to predict the preferential binding site, key amino acid residues involved in ligand binding, and binding affinity of the protein-ligand complexes.

## Supplementary information

Supplementary information

## Data Availability

All data supporting the findings can be found in the results and supplementary sections.
