# Publisher Correction: Atomistic mechanisms underlying the activation of the G protein-coupled sweet receptor heterodimer by sugar alcohol recognition

**DOI:** 10.1038/s41598-019-55307-3

**Published:** 2019-12-09

**Authors:** Panupong Mahalapbutr, Nitchakan Darai, Wanwisa Panman, Aunchan Opasmahakul, Nawee Kungwan, Supot Hannongbua, Thanyada Rungrotmongkol

**Affiliations:** 1 Structural and Computational Biology Research Unit, Department of Biochemistry, Faculty of Science, Chulalongkorn University, Bangkok, 10330 Thailand; 2 Program in Biotechnology, Faculty of Science, Chulalongkorn University, Bangkok, 10330 Thailand; 3 Multidisciplinary Program of Petrochemistry and Polymer Science, Faculty of Science, Chulalongkorn University, Bangkok, 10330 Thailand; 4 Computational Chemistry Center of Excellent, Department of Chemistry, Faculty of Science, Chulalongkorn University, Bangkok, 10330 Thailand; 5 Department of Chemistry, Faculty of Science, Chiang Mai University, Chiang Mai, 50200 Thailand; 6 Center of Excellence in Materials Science and Technology, Chiang Mai University, Chiang Mai, 50200 Thailand; 7 Ph.D. Program in Bioinformatics and Computational Biology, Faculty of Science, Chulalongkorn University, Bangkok, 10330 Thailand; 8 Molecular Sensory Science Center, Faculty of Science, Chulalongkorn University, Bangkok, 10330 Thailand

Correction to: *Scientific Reports* 10.1038/s41598-019-46668-w, published online 15 July 2019

The original version of this article contained a typographical error in the abstract.

“Principal component analysis revealed that the Venus flytrap domain (VFD) of T1R2 monomer was adapted by the induced-fit mechanism to accommodate the focused polyols, in which α-helical residues 233–268 moved significantly closer to stabilize ligands.”

now reads:

“Principal component analysis revealed that the Venus flytrap domain (VFD) of T1R2 monomer was adapted by the induced-fit mechanism to accommodate the focused polyols, in which residues 233–268 moved significantly closer to stabilize ligands.”

Additionally, the original version of this article contained a typographical error in the Results section under the subheading ‘Essential dynamics of the T1R2-T1R3 STR upon polyols complexation’.

“Moreover, binding of these polyols stimulated the direction of motion of α-helical residues 233–268 (purple dashed circle), located near to the binding pocket, to become significantly closer to xylitol and sorbitol, resulting in a compact molecular shape.”

now reads:

“Moreover, binding of these polyols stimulated the direction of motion of residues 233–268 (purple dashed circle), located near to the binding pocket, to become significantly closer to xylitol and sorbitol, resulting in a compact molecular shape.”

The original version of this article also contained a typographical error in the second paragraph of the discussion section.

“Furthermore, binding of the two polyols converted the direction of motion of not only amino acids within a spherical radius of 5 Å but also residues 233–268, which is an α-helix element located near to the binding pocket, to become significantly closer to the ligands.”

now reads:

“Furthermore, binding of the two polyols converted the direction of motion of not only amino acids within a spherical radius of 5 Å but also residues 233–268, which are near to the binding pocket, to become significantly closer to the ligands.”

Furthermore, in Figure 5B the key was incorrect.

“Model 2_MM/PBSA”

now reads

“Model 2_MM/GBSA”.

These errors have now been corrected in the HTML and PDF versions of the article.

Lastly, in Figure 6A, the labelling for the residue N143 was missing. This has now been corrected in the PDF of the article. The HTML was correct at the time of publication.

