# Supplementary material for: Atomistic mechanisms underlying the activation of the G protein-coupled sweet receptor heterodimer by sugar alcohol recognition
Source: Sci Rep. 2019 Jul 15;9:10205. doi: 10.1038/s41598-019-46668-w (PMC6629994; doi:10.1038/s41598-019-46668-w)
Supplement: Supplementary file 1 — Supplementary information [file 41598_2019_46668_MOESM1_ESM.doc]

**Atomistic mechanisms underlying the activation of the G protein-coupled sweet receptor heterodimer by sugar alcohol recognition**

Panupong Mahalapbutr^1^, Nitchakan Darai^2^, Wanwisa Panman^3^, Aunchan Opasmahakul^4^, Nawee Kungwan^5,6^, Supot Hannongbua^4^, Thanyada Rungrotmongkol^1,7,8,*^

*^1^Structural and Computational Biology Research Unit, Department of Biochemistry, Faculty of Science, Chulalongkorn University, Bangkok 10330, Thailand*

*^2^Program in Biotechnology, Faculty of Science, Chulalongkorn University, Bangkok 10330, Thailand*

*^3^Multidisciplinary Program of Petrochemistry and Polymer Science, Faculty of Science, Chulalongkorn University, Bangkok 10330, Thailand*

*^4^Computational Chemistry Center of Excellent, Department of Chemistry, Faculty of Science, Chulalongkorn University, Bangkok 10330, Thailand*

*^5^Department of Chemistry, Faculty of Science, Chiang Mai University, Chiang Mai 50200, Thailand*

*^6^Center of Excellence in Materials Science and Technology, Chiang Mai University, Chiang Mai 50200, Thailand*

*^7^Ph.D. Program in Bioinformatics and Computational Biology, Faculty of Science, Chulalongkorn University, Bangkok 10330, Thailand*

*^8^Molecular Sensory Science Center, Chulalongkorn University, Bangkok 10330, Thailand*

E-mail: thanyada.r@chula.ac.th, [t.rungrotmongkol@gmail.com](mailto:t.rungrotmongkol@gmail.com)

Phone: +66-2218-5426. Fax: +66-2218-5418.

**Supplementary information**


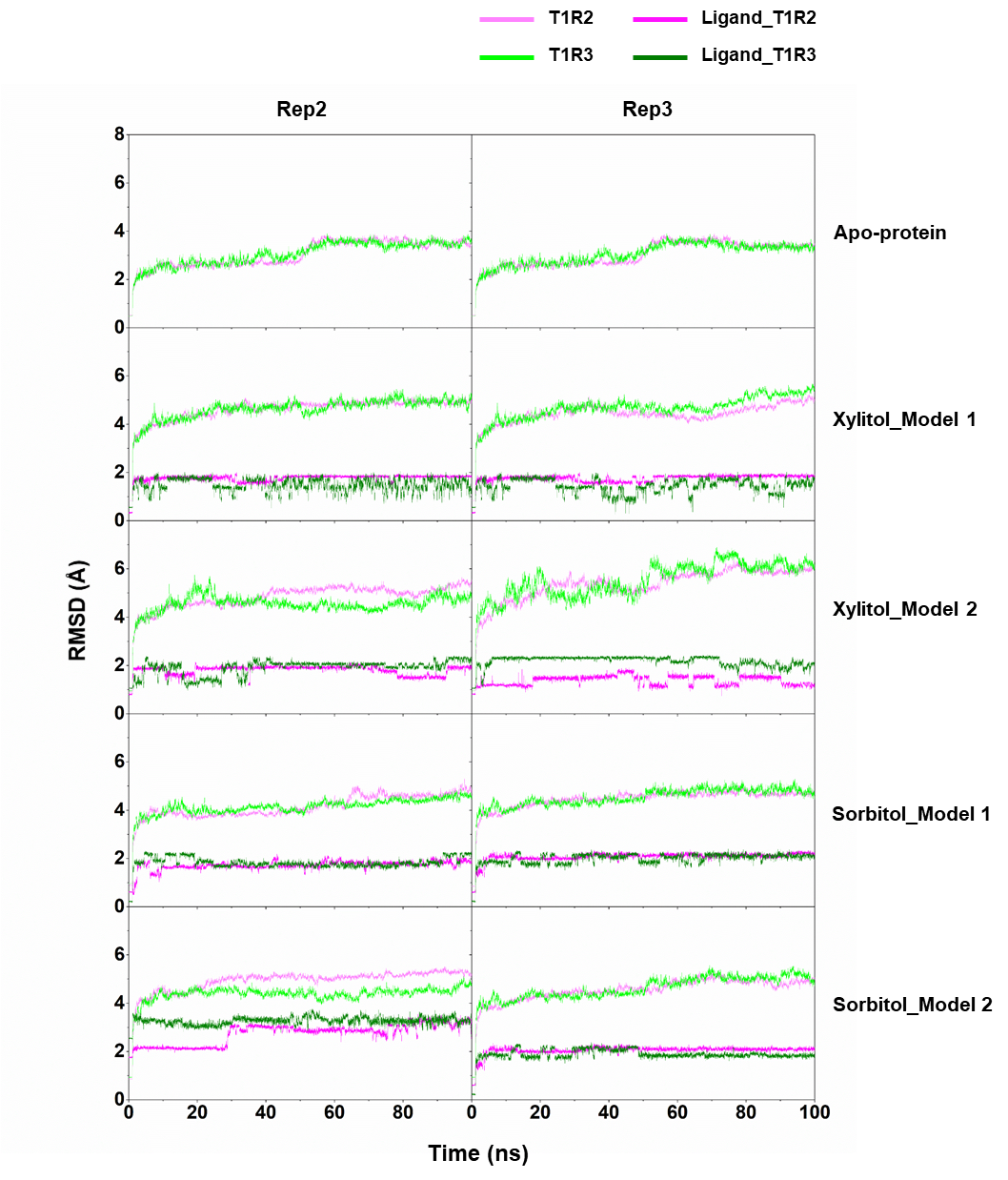


**Figure S1.** RMSD plot of all simulated models.


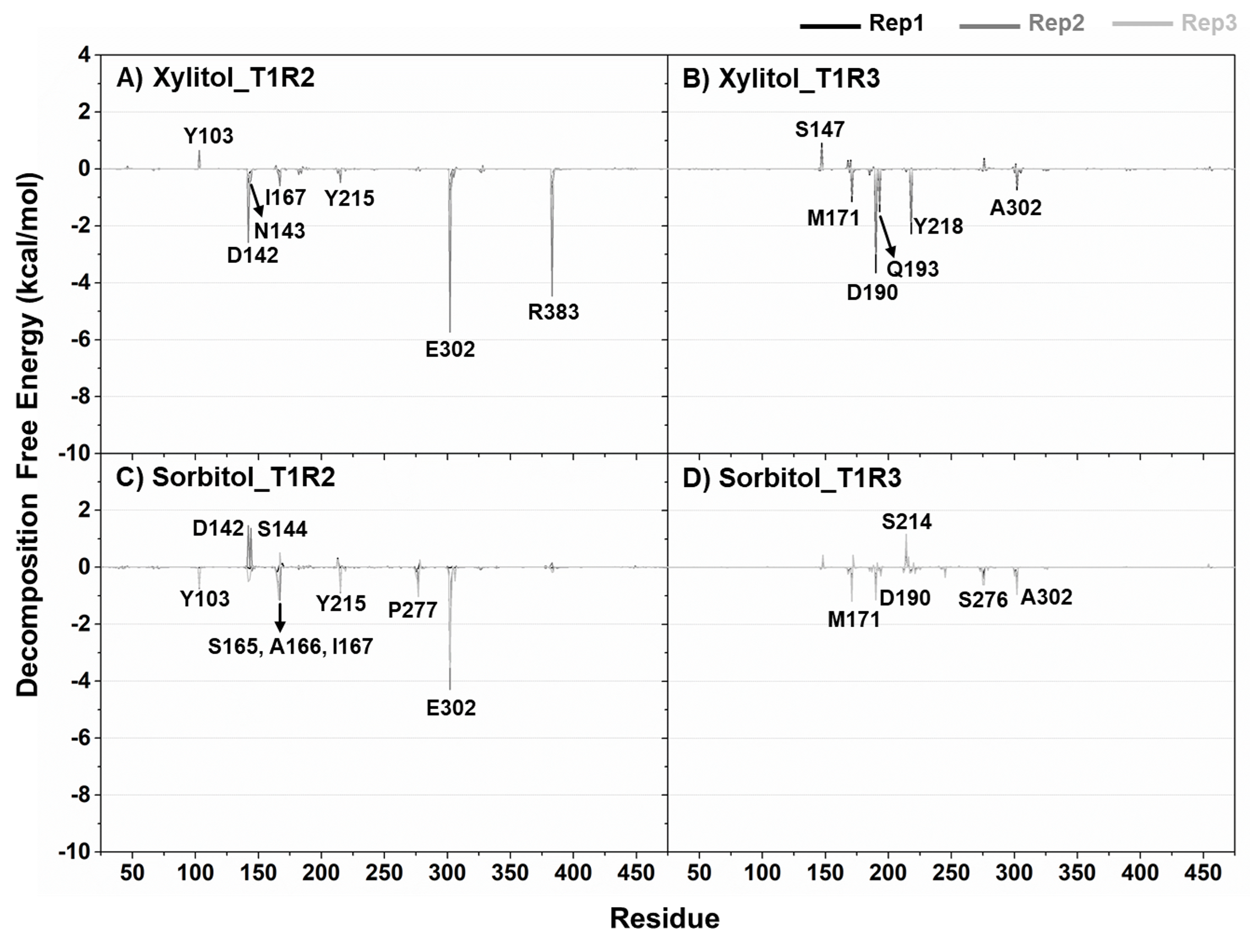


**Figure S2.** The
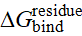
 (kcal/mol) results of model 2 for xylitol and sorbitol bindings to T1R2 (**left**) and T1R3 (**right**) monomers.


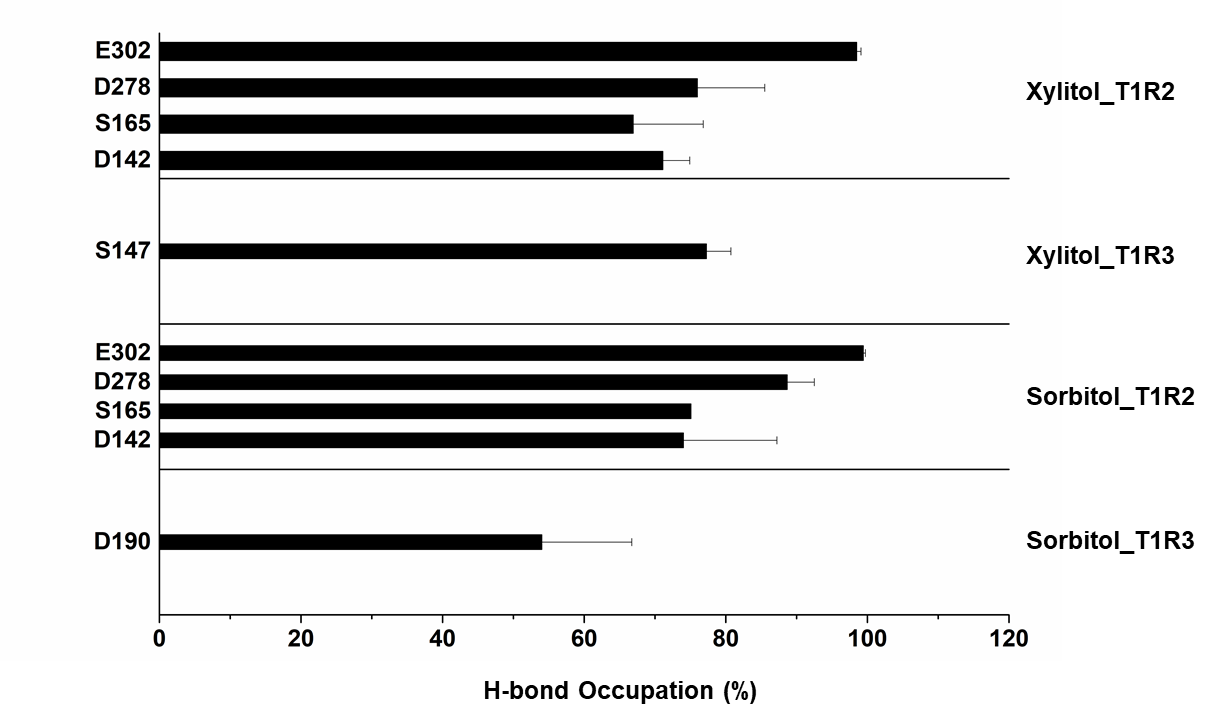


**Figure S3.** H-bond occupation (%) of all simulated complexes (model 2).


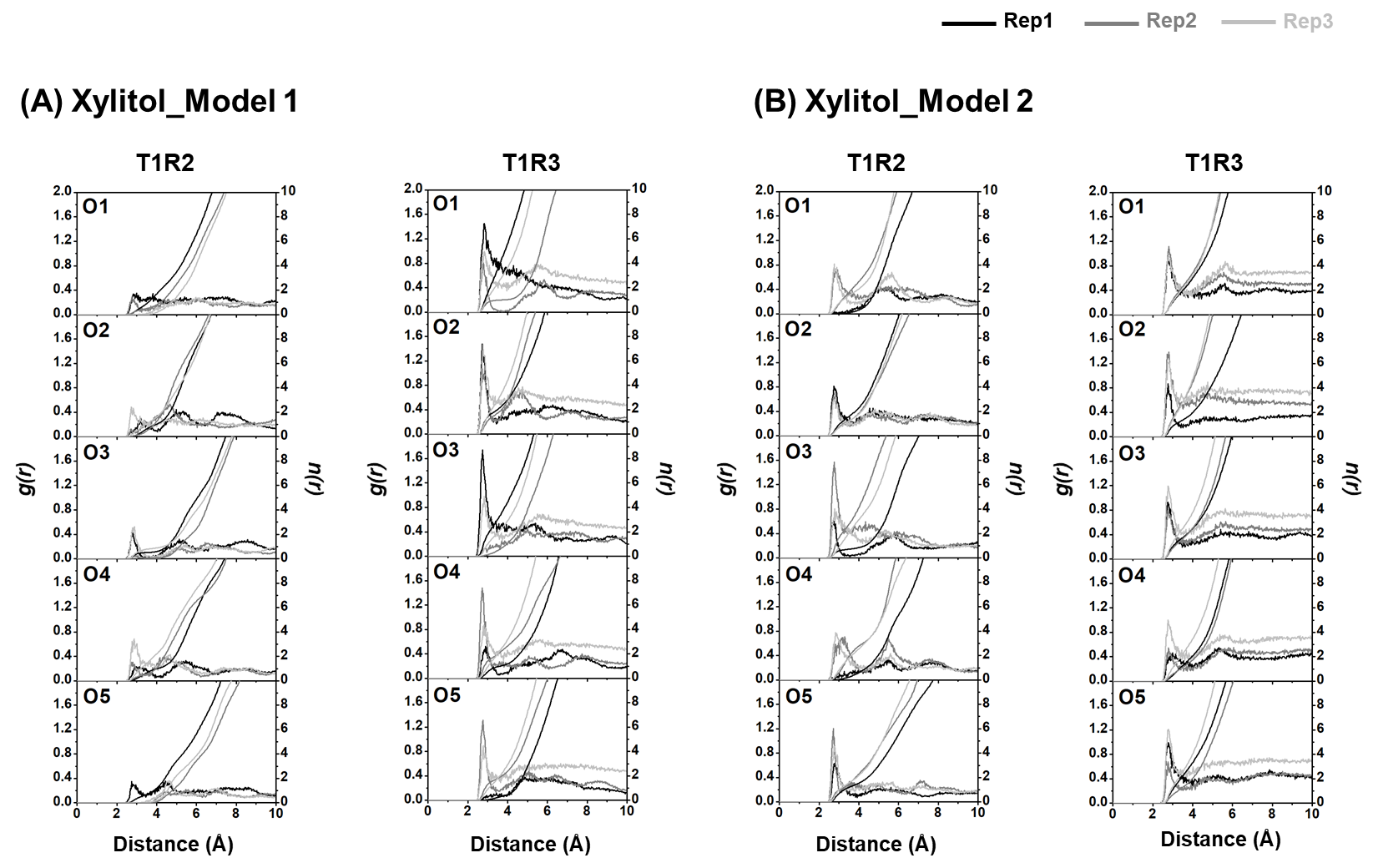


**Figure S4.** RDF plot of xylitol/T1R2-T1R3 complexes taken from three independent MD simulations (MD1-3).


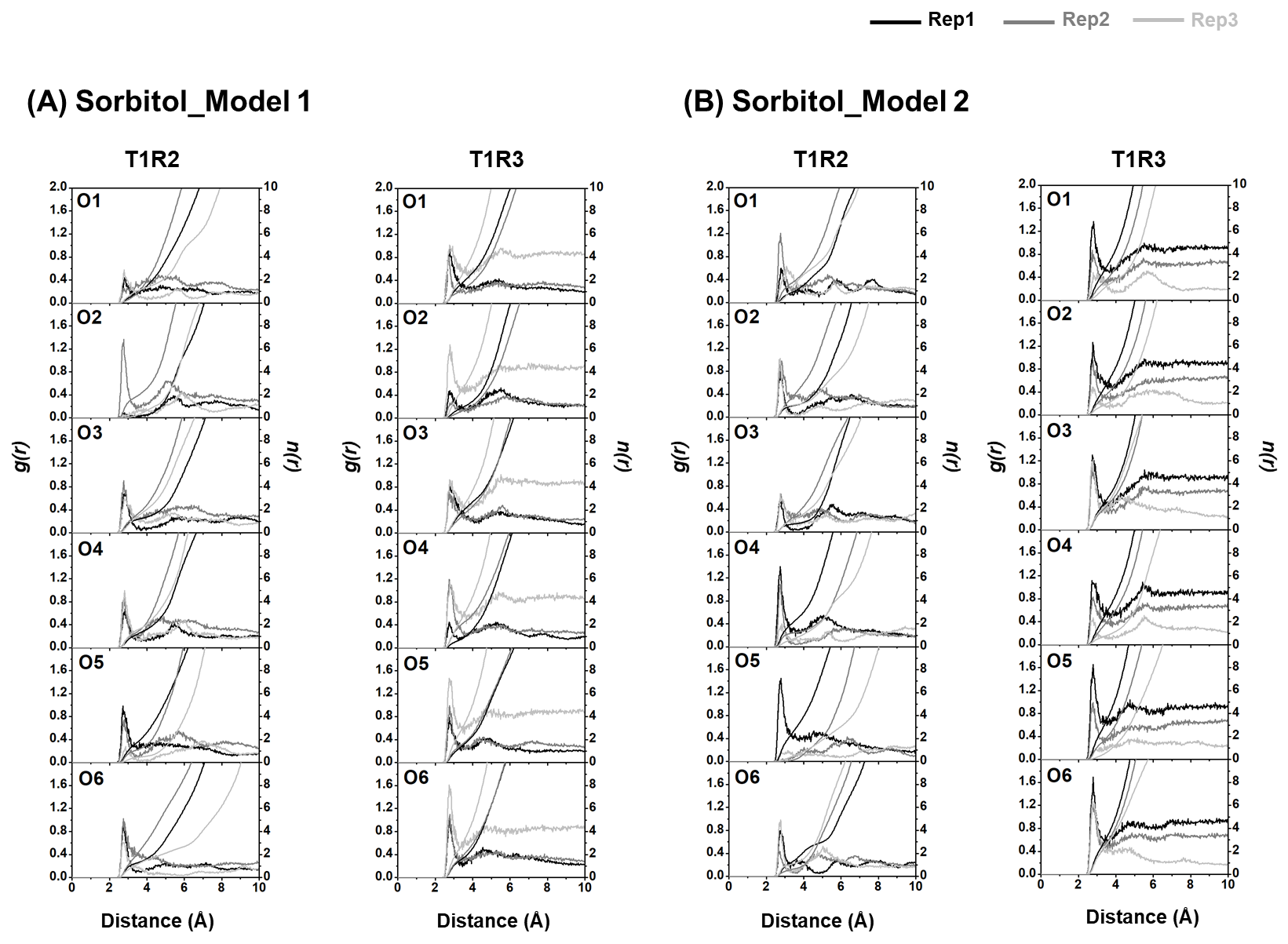


**Figure S5.** RDF plot of sorbitol/T1R2-T1R3 complexes taken from three independent MD simulations (MD1-3).

**Table S1. Docking interaction energy (kcal/mol) obtained from two different programs CDOCKER and FlexX.**

|  | **Docking energy (kcal/mol)** | | | |
| --- | --- | --- | --- | --- |
|  | **CDOCKER** | | **FlexX** | |
|  | **Sorbitol** | **Xylitol** | **Sorbitol** | **Xylitol** |
| **Venus flytrap domain (VFD)** | T1R2 (-40.83)  T1R3 (-30.26) | T1R2 (-35.73)  T1R3 (-29.12) | T1R2 (-18.19)  T1R3 (-18.22) | T1R2 (-17.24)  T1R3 (-13.97) |
| **Cysteine-rich domain (CRD*)** | -25.07 | -27.16 | -10.3 | -7.7 |
| **Transmembrane domain (TMD*)** | 6.32 | -23.84 | -5.2 | -4.6 |

*CRD and TMD structures used for docking were obtained from previous study^1^.

**Table 2**. Atom names, atom types, and partial atomic charges of sorbitol.

**
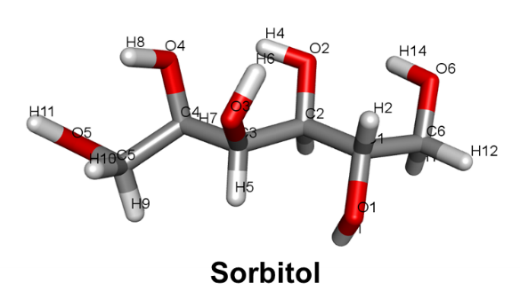
**

| **name** | **type** | **q** |
| --- | --- | --- |
| O5 | OH | -0.702761 |
| H11 | HO | 0.436626 |
| C5 | CT | 0.236548 |
| H9 | H1 | 0.039433 |
| H10 | H1 | 0.039433 |
| C4 | CT | 0.164296 |
| O4 | OH | -0.676495 |
| H8 | HO | 0.451379 |
| H7 | H1 | 0.06831 |
| C3 | CT | 0.151043 |
| O3 | OH | -0.687327 |
| H6 | HO | 0.450021 |
| H5 | H1 | 0.029493 |
| C2 | CT | 0.151043 |
| O2 | OH | -0.687327 |
| H4 | HO | 0.450021 |
| H3 | H1 | 0.029493 |
| C1 | CT | 0.164296 |
| O1 | OH | -0.676495 |
| H1 | HO | 0.451379 |
| H2 | H1 | 0.06831 |
| C6 | CT | 0.236548 |
| H12 | H1 | 0.039433 |
| H13 | H1 | 0.039433 |
| O6 | OH | -0.702761 |
| H14 | HO | 0.436626 |

**Table S3**. Mass, bond, angle, and dihedral angle values of sorbitol.

| **MASS** | | | | |
| --- | --- | --- | --- | --- |
| **MASS** | **mass** | **pol** |  |  |
| OH | 16.000 | 0.465 |  |  |
| HO | 1.008 | 0.135 |  |  |
| CT | 12.010 | 0.878 |  |  |
| H1 | 1.008 | 0.135 |  |  |
| **BOND** | | | | |
| **BOND** | **K(kcal.mol^-1^.ang^-2^)** | **Dist0(ang)** |  |  |
| OH-HO | 369.60 | 0.974 |  |  |
| OH-CT | 314.10 | 1.426 |  |  |
| CT-H1 | 337.30 | 1.092 |  |  |
| CT-CT | 303.10 | 1.535 |  |  |
| **ANGLE** | | | | |
| **ANGLE** | **K(kcal.mol^-1^.ang^-2^)** | **Theta0(deg)** |  |  |
| OH-CT-H1 | 51.07 | 109.50 |  |  |
| OH-CT-CT | 67.72 | 109.43 |  |  |
| HO-OH-CT | 47.09 | 108.16 |  |  |
| CT-CT-H1 | 46.37 | 110.05 |  |  |
| CT-CT-CT | 63.21 | 110.63 |  |  |
| H1-CT-H1 | 39.43 | 108.35 |  |  |
| **DIHEDRAL ANGLE** | | | | |
| **DIHEDRAL** | **Path** | **V(kcal.mol^-1^.rad^-1^)** | **Phase(deg.)** | **Period** |
| OH-CT-CT-OH | 1 | 0.144 | 0 | -3 |
| OH-CT-CT-OH | 1 | 1.175 | 0 | 2 |
| OH-CT-CT-H1 | 1 | 0.000 | 0 | -3 |
| OH-CT-CT-H1 | 1 | 0.250 | 0 | 1 |
| OH-CT-CT-CT | 1 | 0.156 | 0 | 3 |
| HO-OH-CT-H1 | 1 | 0.167 | 0 | 3 |
| HO-OH-CT-CT | 1 | 0.160 | 0 | -3 |
| HO-OH-CT-CT | 1 | 0.250 | 0 | 1 |
| CT-CT-CT-H1 | 1 | 0.160 | 0 | 3 |
| CT-CT-CT-CT | 1 | 0.180 | 0 | -3 |
| CT-CT-CT-CT | 1 | 0.250 | 180 | -2 |
| CT-CT-CT-CT | 1 | 0.200 | 180 | 1 |
| H1-CT-CT-H1 | 1 | 0.150 | 0 | 3 |

**Table S4**. Atom names, atom types, and partial atomic charges of xylitol.

**
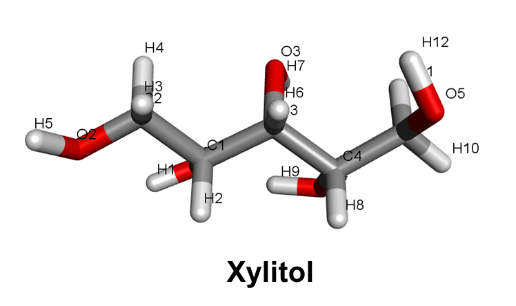
**

| **name** | **type** | **q** |
| --- | --- | --- |
| O2 | OH | -0.690811 |
| H5 | HO | 0.422611 |
| C2 | CT | 0.291111 |
| H3 | H1 | 0.017115 |
| H4 | H1 | 0.017115 |
| C1 | CT | 0.176318 |
| O1 | OH | -0.652181 |
| H1 | HO | 0.438662 |
| H2 | H1 | 0.036863 |
| C3 | CT | -0.047942 |
| O3 | OH | -0.646670 |
| H7 | HO | 0.459907 |
| H6 | H1 | 0.121098 |
| C4 | CT | 0.176318 |
| O4 | OH | -0.652181 |
| H9 | HO | 0.438662 |
| H8 | H1 | 0.036863 |
| C5 | CT | 0.291111 |
| H10 | H1 | 0.017115 |
| H11 | H1 | 0.017115 |
| O5 | OH | -0.690811 |
| H12 | HO | 0.422611 |

**Table S5**. Mass, bond, angle, and dihedral angle values of xylitol.

| **MASS** | | | | |
| --- | --- | --- | --- | --- |
| **MASS** | **mass** | **pol** |  |  |
| OH | 16.000 | 0.465 |  |  |
| HO | 1.008 | 0.135 |  |  |
| CT | 12.010 | 0.878 |  |  |
| H1 | 1.008 | 0.135 |  |  |
| **BOND** | | | | |
| **BOND** | **K(kcal.mol^-1^.ang^-2^)** | **Dist0(ang)** |  |  |
| OH-HO | 369.60 | 0.974 |  |  |
| OH-CT | 314.10 | 1.426 |  |  |
| CT-H1 | 337.30 | 1.092 |  |  |
| CT-CT | 303.10 | 1.535 |  |  |
| **ANGLE** | | | | |
| **ANGLE** | **K(kcal.mol^-1^.ang^-2^)** | **Theta0(deg)** |  |  |
| OH-CT-H1 | 51.07 | 109.50 |  |  |
| OH-CT-CT | 67.72 | 109.43 |  |  |
| HO-OH-CT | 47.09 | 108.16 |  |  |
| CT-CT-H1 | 46.37 | 110.05 |  |  |
| CT-CT-CT | 63.21 | 110.63 |  |  |
| H1-CT-H1 | 39.43 | 108.35 |  |  |
| **DIHEDRAL ANGLE** | | | | |
| **DIHEDRAL** | **Path** | **V(kcal.mol^-1^.rad^-1^)** | **Phase(deg.)** | **Period** |
| OH-CT-CT-OH | 1 | 0.144 | 0 | -3 |
| OH-CT-CT-OH | 1 | 1.175 | 0 | 2 |
| OH-CT-CT-H1 | 1 | 0.000 | 0 | -3 |
| OH-CT-CT-H1 | 1 | 0.250 | 0 | 1 |
| OH-CT-CT-CT | 1 | 0.156 | 0 | 3 |
| HO-OH-CT-H1 | 1 | 0.167 | 0 | 3 |
| HO-OH-CT-CT | 1 | 0.160 | 0 | -3 |
| HO-OH-CT-CT | 1 | 0.250 | 0 | 1 |
| CT-CT-CT-H1 | 1 | 0.160 | 0 | 3 |
| CT-CT-CT-CT | 1 | 0.180 | 0 | -3 |
| CT-CT-CT-CT | 1 | 0.250 | 180 | -2 |
| CT-CT-CT-CT | 1 | 0.200 | 180 | 1 |
| H1-CT-CT-H1 | 1 | 0.150 | 0 | 3 |

**Reference**

1 Chéron, J.-B., Golebiowski, J., Antonczak, S. & Fiorucci, S. The anatomy of mammalian sweet taste receptors. 85, 332-341, doi:10.1002/prot.25228 (2017).
